# A centenary for bacterial spot of tomato and pepper

**DOI:** 10.1111/mpp.13125

**Published:** 2021-09-02

**Authors:** Ebrahim Osdaghi, Jeffrey B. Jones, Anuj Sharma, Erica M. Goss, Peter Abrahamian, Eric A. Newberry, Neha Potnis, Renato Carvalho, Manoj Choudhary, Mathews L. Paret, Sujan Timilsina, Gary E. Vallad

**Affiliations:** ^1^ Department of Plant Protection College of Agriculture University of Tehran Karaj Iran; ^2^ Plant Pathology Department University of Florida Gainesville Florida USA; ^3^ Emerging Pathogens Institute University of Florida Gainesville Florida USA; ^4^ Gulf Coast Research and Education Center University of Florida Wimauma Florida USA; ^5^ Department of Entomology and Plant Pathology Auburn University Auburn Alabama USA; ^6^ Department of Plant Pathology North Florida Research and Education Center University of Florida Quincy Florida USA

**Keywords:** *Capsicum annuum*, Solanaceae, *Solanum lycopersicum*, *Xanthomonas euvesicatoria* pv. *euvesicatoria*, *Xanthomonas euvesicatoria* pv. *perforans*, *Xanthomonas hortorum* pv. *gardneri*, *Xanthomonas vesicatoria*

## Abstract

**Disease symptoms:**

Symptoms include water‐soaked areas surrounded by chlorosis turning into necrotic spots on all aerial parts of plants. On tomato fruits, small, water‐soaked, or slightly raised pale‐green spots with greenish‐white halos are formed, ultimately becoming dark brown and slightly sunken with a scabby or wart‐like surface.

**Host range:**

Main and economically important hosts include different types of tomatoes and peppers. Alternative solanaceous and nonsolanaceous hosts include *Datura* spp., *Hyoscyamus* spp., *Lycium* spp., *Nicotiana rustica*, *Physalis* spp., *Solanum* spp., *Amaranthus lividus*, *Emilia fosbergii*, *Euphorbia heterophylla*, *Nicandra physaloides*, *Physalis pubescens*, *Sida glomerata*, and *Solanum americanum*.

**Taxonomic status of the pathogen:**

Domain, Bacteria; phylum, *Proteobacteria*; class, *Gammaproteobacteria*; order, *Xanthomonadales*; family, *Xanthomonadaceae*; genus, *Xanthomonas*; species, *X*. *euvesicatoria*, *X*. *hortorum*, *X*. *vesicatoria*.

**Synonyms (nonpreferred scientific names):**

*Bacterium exitiosum*, *Bacterium vesicatorium*, *Phytomonas exitiosa*, *Phytomonas vesicatoria*, *Pseudomonas exitiosa*, *Pseudomonas gardneri*, *Pseudomonas vesicatoria*, *Xanthomonas axonopodis* pv. *vesicatoria*, *Xanthomonas campestris* pv. *vesicatoria*, *Xanthomonas cynarae* pv. *gardneri*, *Xanthomonas gardneri*, *Xanthomonas perforans*.

**Microbiological properties:**

Colonies are gram‐negative, oxidase‐negative, and catalase‐positive and have oxidative metabolism. Pale‐yellow domed circular colonies of 1–2 mm in diameter grow on general culture media.

**Distribution:**

The bacteria are widespread in Africa, Brazil, Canada and the USA, Australia, eastern Europe, and south‐east Asia. Occurrence in western Europe is restricted.

**Phytosanitary categorization:**

A2 no. 157, EU Annex designation II/A2.

**EPPO codes:**

XANTEU, XANTGA, XANTPF, XANTVE.

## Graphical abstract

In this review we provide a historical perspective as well as an updated overview on the aetiology, epidemiology, and management strategies of bacterial spot of tomato and pepper.

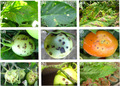

## 1 INTRODUCTION

Bacterial spot of tomato and pepper caused by four distinct *Xanthomonas* lineages, *Xanthomonas euvesicatoria* pv. *euvesicatoria*, *X*. *euvesicatoria* pv. *perforans*, *Xanthomonas hortorum* pv. *gardneri*, and *Xanthomonas vesicatoria*, is an economically important disease threatening the pepper and tomato industry around the globe (Potnis et al., 2015). Due to the seedborne nature of the pathogens, management of the disease has been a major problem since its original description in 1920 (Doidge, 1920). As a complex disease caused by a set of heterogeneous xanthomonads, bacterial spot occurs in many countries on greenhouse‐grown as well as field‐grown tomatoes with a particular importance in the areas characterized by warm and humid conditions (EPPO, 2013). In the case of severe infections, direct losses of 23%–44% could occur in fruit yield while indirect losses in severely infected plants are mainly due to shedding of leaves and exposure of fruits to sunlight, leading to sunscald (Bashan et al., 1985). The causal agents are included in the A2 (high risk) list of quarantine pathogens of the European and Mediterranean Plant Protection Organization (EPPO codes: XANTEU, XANTGA, XANTPF, XANTVE; A2 no. 157, EU Annex designation II/A2). Hence, they are under strict quarantine control and zero tolerance all over the globe (EPPO, 2013; EFSA 2014). Due to the taxonomic complexities among the bacterial spot xanthomonads, classification of the four lineages has changed several times over the past couple of decades. More specifically, since 2016, the taxonomic position of three of the four bacterial spot xanthomonads has changed, complicating pathogen identification and lineage/species determination (Constantin et al., 2016; Morinière et al., 2020). Furthermore, within the past few years, predominance of high‐throughput genome sequencing technologies paved the way for research on complete genome resources, which led to a deeper understanding of genetic diversity, pathogenicity mechanisms, and genomic repertories of the bacterial spot pathogens (Timilsina et al., 2020).

In this review, first we provide a brief taxonomic history and an updated overview on the classification and taxonomic position of the four bacterial spot xanthomonads. Then, the recombination‐driven population structure of the pathogens as revealed by the whole genome sequence data within the past few years is described. Furthermore, new findings with respect to the pathogenicity mechanisms and virulence properties of the bacterial spot xanthomonads are reviewed, highlighting the role of type III secretion effectors (T3Es) that include transcription activator‐like effectors (TALE) and non‐TAL effectors. In this regard, Potnis et al. (2015) have recently reviewed basic molecular aspects of the host–pathogen interactions; hence, we mainly concentrate on the complete genome sequence‐based achievements in the past few years. Finally, as bacterial resistance to copper‐based bactericides has become evident around the globe (Lamichhane et al., 2018), we provide a list of new noncopper‐based available options introduced within the past decade to combat bacterial spot disease in the 21st century's tomato and pepper industry.

## 2 UPDATE ON TAXONOMY OF THE PATHOGENS

Bacterial spot of tomato and pepper was reported for the first time almost simultaneously in South Africa and the USA (Indiana) in the early 1920s. The causal agent was named *Bacterium vesicatorium* (Doidge, 1920, 1921; Gardner & Kendrick, 1921, 1923), which later changed to *X. vesicatoria* (Dowson, 1939). In 1978, the taxonomic status of several species within *Xanthomonas* was subsided into the “pathovar” level and the tomato and pepper pathogen was reclassified as *Xanthomonas campestris* pv. *vesicatoria* (Young et al., 1978). During the 1990s, it was shown that two genetically distinct groups existed within *X*. *campestris* pv. *vesicatoria*, and the two groups were designated as group A, including the strains resembling those isolated in South Africa, and group B, including the strains resembling those isolated in the USA (Stall et al., 1994). Furthermore, a third group of amylolytic and pectolytic strains was isolated from tomato in Florida and designated as group C (Jones et al., 1995). Subsequently, Vauterin et al. (1995) reclassified all the xanthomonads and divided the *X*. *campestris* pv. *vesicatoria* members into two species, *Xanthomonas axonopodis* pv. *vesicatoria* (group A and C strains) and *X*. *vesicatoria* (group B strains). On the other hand, a bacterial pathogen was isolated from tomato in the former Yugoslavia in 1957 and, despite its yellow‐pigmented colonies, the causal agent was originally named as *Pseudomonas gardneri* (Šutic, 1957). The pathogen was later proposed to be transferred to *Xanthomonas* and considered as group D of the bacterial spot pathogens (Dye, 1966; Jones et al., 2000; de Vos et al., 1985). In 2004, DNA:DNA hybridization analyses showed that *X*. *axonopodis* pv. *vesicatoria* group A and C strains have less than 70% DNA relatedness with each other, with the type strain of *X*. *axonopodis*, and with the other species within *Xanthomonas*. The group A strains closely resembled strains originally isolated by Doidge in South Africa in 1920, while the group C strains were originally isolated from tomato in Florida in the early 1990s. Hence, the tomato‐ and pepper‐pathogenic xanthomonads were reclassified within four stand‐alone species, *X*. *euvesicatoria* (group A), *X*. *vesicatoria* (group B), *Xanthomonas perforans* (group C), and *Xanthomonas gardneri* (group D) (Jones et al., 2004, 2006).

**FIGURE 1:**
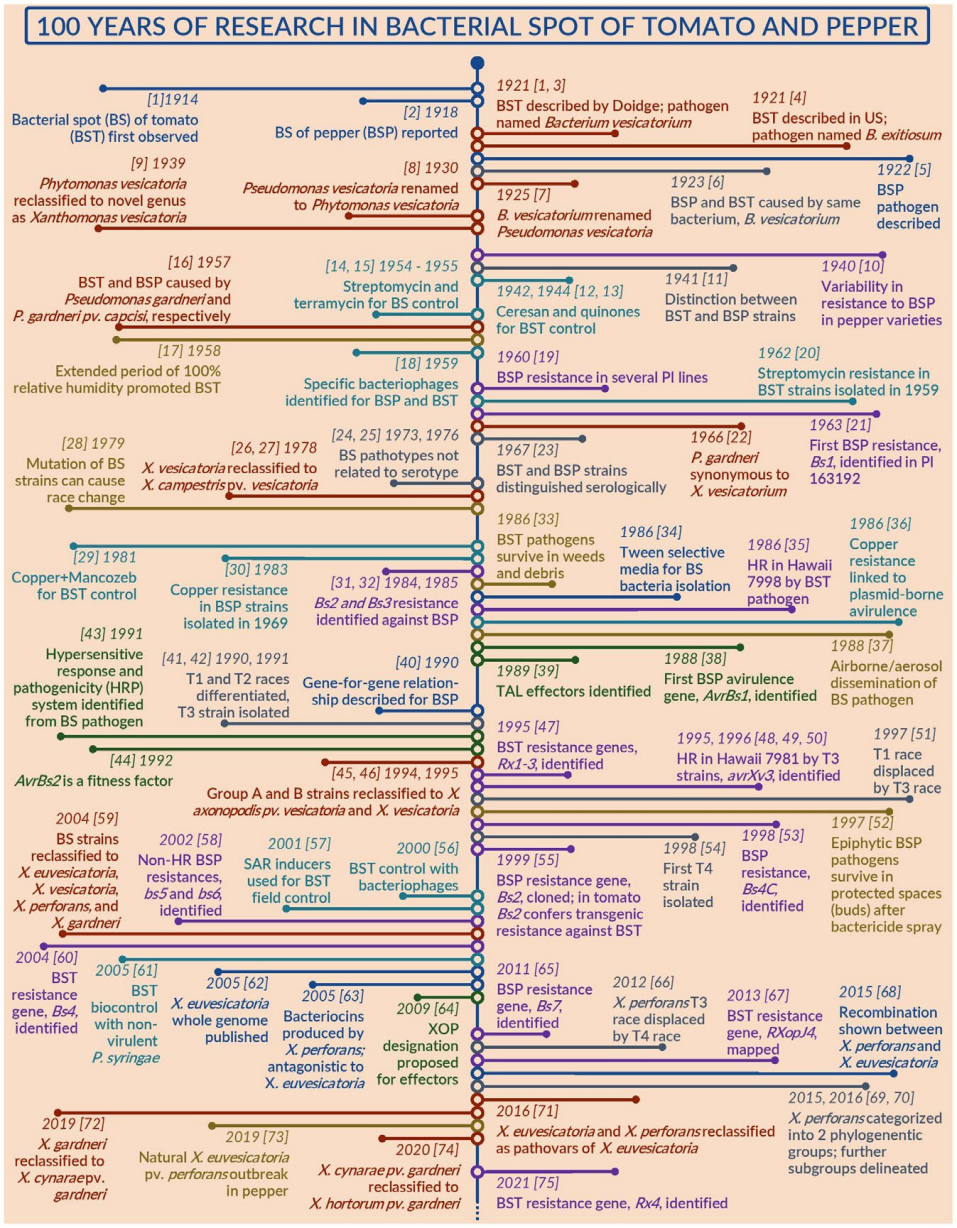
A century‐wide timeline of major milestones in the study of bacterial spot of tomato and pepper. A continuous set of rings along the centre represents one decade. The colour coding represents the following: blue, general; red, taxonomy; grey, strain classification; green, effectors; purple, races and host resistance; yellow, epidemiology; and cyan, disease management. The sources of data for each event are shown within the brackets and the numbers represent the following: [1] Doidge (1920); [2] Sherbakoff (1918); [3] Doidge (1921); [4] Gardner and Kendrick (1921); [5] Higgins (1922); [6] Gardner and Kendrick (1923); [7] Stevens (1925); [8] Bergey et al. (1930); [9] Dowson (1939); [10] Horsfall et al. (1940); [11] Burkholder and Li (1941); [12] Beattie et al. (1942); [13] Nagel (1944); [14] Conover (1954); [15] Conover (1955); [16] Šutic (1957); [17] Davis and Halmos (1958); [18] Klement (1959); [19] Sowell (1960); [20] Stall and Thayer (1962); [21] Cook and Stall (1963); [22] Dye (1966); [23] O’Brien et al. (1967); [24] Charudattan et al. (1973); [25] Schaad (1976); [26] Young et al. (1978); [27] Dye et al. (1980); [28] Dahlbeck and Stall (1979); [29] Conover and Gerhold (1981); [30] Marco and Stall (1983); [31] Cook and Guevara (1984); [32] Kim and Hartmann (1985); [33] Jones et al. (1986); [34] McGuire et al. (1986); [35] Jones and Scott (1986); [36] Stall et al. (1986); [37] McInnes et al. (1988); [38] Ronald and Staskawicz (1988); [39] Bonas et al. (1989); [40] Minsavage et al. (1990); [41] Wang et al. (1990); [42] Jones et al. (1995); [43] Bonas et al. (1991); [44] Kearney and Staskawicz (1990); [45] Stall et al. (1994); [46] Vauterin et al. (1995); [47] Yu et al. (1995); [48] Scott et al. (1995); [49] Scott et al. (1966); [50] Minsavage et al. (1996); [51] Jones et al. (1998); [52] Pernezny and Collins (1997); [53] Sahin and Miller (1998); [54] Astua‐Monge et al. (2000); [55] Tai et al. (1999); [56] Flaherty et al. (2000); [57] Louws et al. (2001); [58] Jones et al. (2002); [59] Jones et al. (2004); [60] Schornack et al. (2004); [61] Byrne et al. (2005); [62] Thieme et al. (2005); [63] Hert et al. (2005); [64] White et al. (2009); [65] Potnis et al. (2011); [66] Horvath et al. (2012); [67] Sharlach et al. (2013); [68] Timilsina et al. (2015); [69] Schwartz et al. (2015); [70] Timilsina et al. (2016); [71] Constantin et al. (2016); [72] Timilsina, Kara, et al. (2019); [73] Newberry et al. (2019); [74] Morinière et al. (2020); [75] Zhang et al. (2012)

Later, multilocus sequence analysis (MLSA) and typing (MLST) showed that the species *X*. *euvesicatoria*, *X*. *perforans*, and *Xanthomonas alfalfae* were not clearly differentiated as stand‐alone species (Timilsina et al., 2015; Yaripour et al., 2018; Young et al., 2008), which then was confirmed by whole genome sequence‐based phylogenomics (Barak et al., 2016). Hence, the two species *X*. *euvesicatoria* and *X*. *perforans* were reclassified as pathovars of the same species as *X*. *euvesicatoria* pv. *euvesicatoria* and *X*. *euvesicatoria* pv. *perforans*, respectively (Constantin et al., 2016). As for *X*. *gardneri*, the whole genome sequence‐based average nucleotide identity index between representative strains of *X*. *gardneri* and the artichoke (*Cynara cardunculus*) pathogen *Xanthomonas cynarae* was well above the threshold of 95%–96% (Timilsina, Kara, et al., 2019). Hence, *X*. *gardneri* was reclassified as a later heterotypic synonym of *X*. *cynarae* and named *X*. *cynarae* pv. *gardneri* (Timilsina, Kara, et al., 2019). Further comprehensive complete genome sequence‐based investigations showed that *X*. *cynarae* itself is a later heterotypic synonym of the garden crops' pathogen *X*. *hortorum*, thus the tomato pathogen was reclassified as *X*. *hortorum* pv. *gardneri* (Morinière et al., 2020). Taken together, tomato and pepper‐pathogenic xanthomonads are currently classified into four lineages within three species: *X*. *euvesicatoria* pv. *euvesicatoria*, *X*. *euvesicatoria* pv. *perforans*, *X*. *hortorum* pv. *gardneri*, and *X*. *vesicatoria* (Constantin et al., 2016; Morinière et al., 2020). Figure 1 shows a century‐wide overview and timeline of major milestones in the study of bacterial spot of tomato and pepper.

## 3 DISEASE SYMPTOMS

**FIGURE 2:**
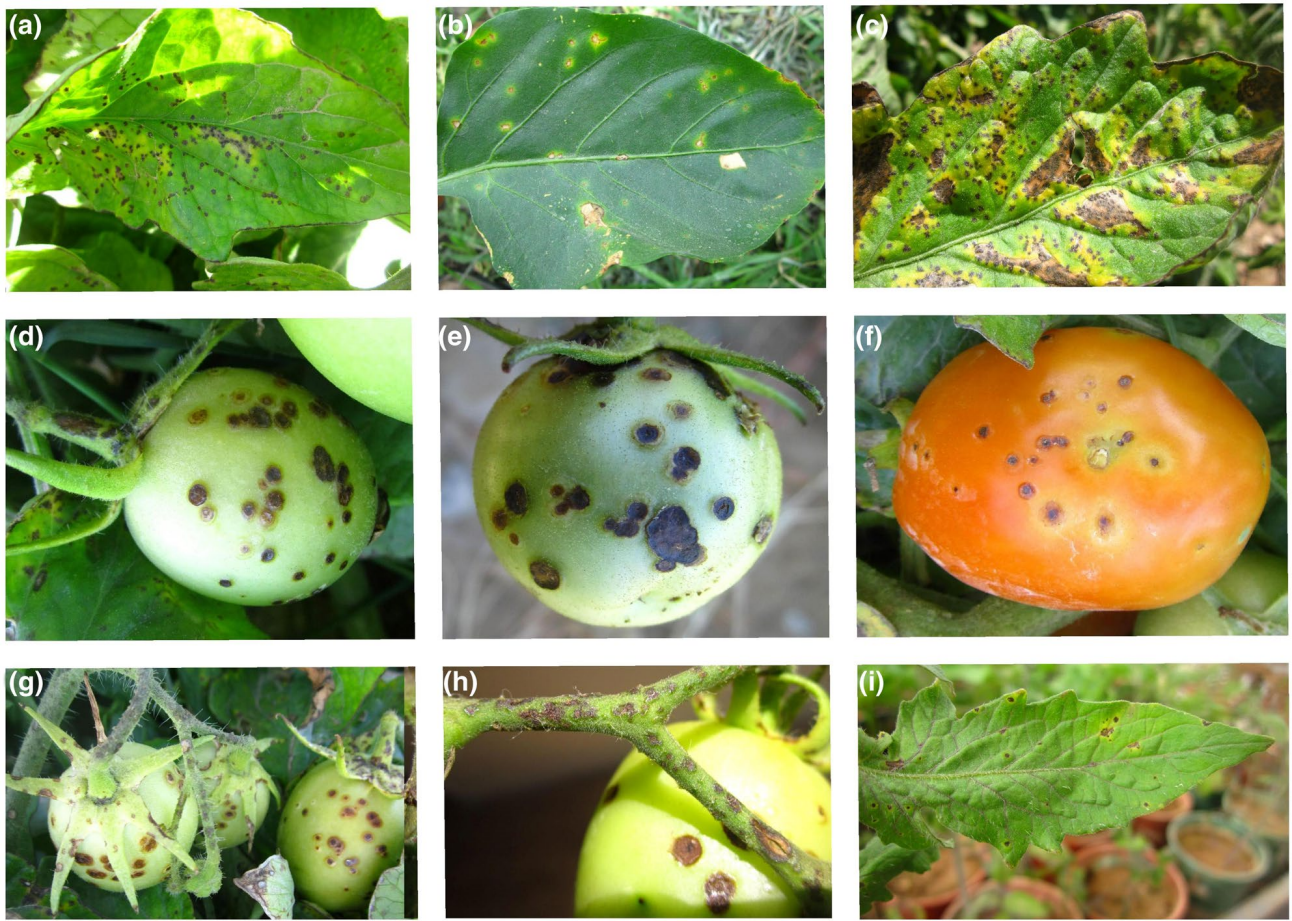
Field symptoms of bacterial spot of tomato and pepper. (a, b) Initial symptoms include water‐soaked spots and/or dark‐brown and greasy lesions on tomato (a) and pepper (b) leaves. (c) Lesions on the leaves with severe infection coalesce giving a blighted appearance. (d, e) On unripe fruits, symptoms include small and water‐soaked spots with greenish‐white halos (d), subsequently becoming dark brown with a scabby or wart‐like surface (e). (f) Ripened fruits with symptoms are unmarketable because of brown lesions and poor quality. (g, h) Brown lesions leading to necrotic areas are also observed on sepals (g), while stem lesions are narrow and elongated up to 5 mm (h). (i) Artificial inoculation of tomato leaf leads to water‐soaked lesions turning into chlorotic and eventually necrotic spots within 12–15 days after inoculation

Bacterial spot symptoms include lesions that initially appear to be water‐soaked and often surrounded by chlorosis that eventually develop into necrotic spots on all aerial parts of plants (Figure 2a–i). Leaf symptoms include small water‐soaked lesions that turn brown and irregular in appearance until becoming dark‐brown and greasy. Water‐soaked appearance is more readily observed in transplant nurseries as well as in the fields where sprinkler irrigation prevails (Figure 2a,b). Leaf lesions may enlarge up to 2–3 mm in diameter while leaves bearing many coalesced lesions have a blighted appearance (Figure 2c). Unripe tomato fruits bear small, water‐soaked or slightly raised pale‐green spots with greenish‐white halos, ultimately becoming dark brown and slightly sunken with a scabby or wart‐like surface (Figure 2d–f). Symptoms on tomato sepals include brown lesions leading to necrotic areas, while stem lesions are narrow and elongated (<3 mm in diameter) and become light brown and rough in appearance over time (Figure 2g,h; Osdaghi et al., 2017). Pith necrosis symptoms caused by *X*. *euvesicatoria* pv. *perforans* have also been recorded on greenhouse‐grown tomatoes in Italy (Aiello et al., 2013). On pepper plants, initial symptoms consist of circular water‐soaked lesions later becoming dark brown to black surrounded by a chlorotic halo, while no shot‐hole appearance is observed (Figure 2b; Osdaghi et al., 2016). Although leaf drop and defoliation are infrequent in tomato plants, in the case of severe infections in pepper, the necrotic spots coalesce, leading to defoliation of the infected leaves, which will probably result in sunscald of the fruits on hot and sunny days. Following artificial inoculation, initial water‐soaked lesions gradually turn into chlorotic and finally necrotic spots on the leaves within 12–15 days after inoculation (Figure 2i).

## 4 HOST RANGE OF THE PATHOGENS

The host range of the bacterial spot pathogens expands over different types of tomatoes (table fruit and processing tomato [*Solanum lycopersicum*], cherry tomato [*S*. *lycopersicum* var. *cerasiforme*], and currant tomato [*Solanum pimpinellifolium*]) as well as peppers (*Capsicum annuum*, *Capsicum frutescens*, *Capsicum baccatum*, *Capsicum anomalum*, *Capsicum chinensis*, and *Capsicum pubescens*) (Jones et al., 1998; Stall et al., 2009). Various pepper and tomato races of the bacterial spot pathogens are reported as being pathogenic only on tomato, only on pepper, or on both tomato and pepper (Jones et al., 1995; Kebede et al., 2014; Potnis et al., 2015; Stall et al., 2009). *X. hortorum* pv. *gardneri* and *X*. *euvesicatoria* pv. *euvesicatoria* are reported as pathogens of both tomato and pepper, while *X*. *vesicatoria* strains primarily infect tomato and until recently *X*. *euvesicatoria* pv. *perforans* strains have only been isolated from tomato (Timilsina et al., 2015). However, during the past several years a host range shift in the *X*. *euvesicatoria* pv. *perforans* population has been observed around the globe. In 2010, an *X*. *euvesicatoria* pv. *perforans* strain was isolated from a pepper field in Florida, suggesting a recent host range expansion (Potnis et al., 2015). Furthermore, Newberry et al. (2019) isolated *X*. *euvesicatoria* pv. *perforans* strains from pepper plants with symptoms in Alabama (USA). A wide range of plants belonging to the Solanaceae family (*Datura* spp., *Hyoscyamus* spp., *Lycium* spp., *Nicotiana rustica*, *Physalis* spp., and *Solanum* spp.) have been recorded as incidental hosts of the bacterial spot xanthomonads (EPPO, 2013).

Nonsolanaceous plant species have also been reported to be alternative hosts of the bacterial spot pathogens. For instance, *X*. *euvesicatoria* pv. *euvesicatoria* was isolated from *Aeollanthus suaveolens*, *Amaranthus lividus*, *Sida glomerata*, and *Emilia fosbergii* in Brazil (Santos et al., 2020). The bacterium also causes bacterial spot on *Physalis pubescens* in north‐east China (Song et al., 2019). *X. hortorum* pv. *gardneri* was isolated from *Euphorbia heterophylla* plants naturally grown among tomato plants in commercial fields showing leaf lesion symptoms in Brazil. The *X*. *hortorum* pv. *gardneri* strains isolated from *Euphorbia heterophylla* were capable of inducing leaf spot symptoms on *Nicandra physaloides* and *Solanum americanum* species (Araújo et al., 2015). Furthermore, artificial inoculation of *X*. *hortorum* pv. *gardneri* strains on artichoke leaves caused mild disease symptoms (Kara et al., 2018; Tilimisina, Kara, et al., 2019). *X. euvesicatoria* pv. *perforans* has also been associated with bacterial blight and dieback of *Eucalyptus pellita* seedlings in Indonesia (Bophela et al., 2019).

## 5 BACTERIOLOGICAL FEATURES OF THE PATHOGENS

The bacterial spot xanthomonads produce pale‐yellow, domed circular colonies on general culture media, for example, nutrient agar (NA), yeast extract‐peptone‐glucose agar, and yeast extract‐dextrose‐CaCO_3_ agar, that are 1–2 mm in diameter after 2–3 days of incubation at 22–27 °C (Schaad et al., 2001). The bacteria are gram‐negative, oxidase‐negative, and catalase‐positive and have oxidative metabolism while they do not grow on 0.1% triphenyl tetrazolium chloride (TTC). Strains of *X*. *euvesicatoria* pv. *perforans* and *X*. *vesicatoria* possess strong amylolytic and pectolytic activity, whereas *X*. *euvesicatoria* pv. *euvesicatoria* and *X*. *hortorum* pv. *gardneri* are, in general, weakly amylolytic or nonamylolytic and nonpectolytic (Jones et al., 2004). However, atypical amylolytic and pectolytic strains of *X*. *euvesicatoria* pv. *euvesicatoria* are increasingly being reported around the globe (Bouzar et al., 1996; Jibrin et al., 2015).

## 6 GENETIC DIVERSITY AND POPULATION STRUCTURE

Bacterial spot xanthomonads consist of a group of taxonomically heterogeneous lineages belonging to different species/pathovars. However, dramatic changes in the dominant lineages and population structure in a local area have been documented during the past few decades. For instance, before 1991, *X*. *euvesicatoria* pv. *euvesicatoria* was the only causal agent of the disease in Florida, while this taxon was entirely replaced on tomato over the course of about 15 years (Horvath et al., 2012). This was further shown in a more recent survey in 2017 where all 585 strains collected in 70 tomato fields in the state were identified as *X*. *euvesicatoria* pv. *perforans* (Klein‐Gordon et al., 2021). A possible explanation for this displacement is that *X*. *euvesicatoria* pv. *perforans* strains produce inhibitory bacteriocins that target strains of *X*. *euvesicatoria* pv. *euvesicatoria* (Hert et al., 2009), although some contemporary strains have now lost this ability (Klein‐Gordon et al., 2021). Similar changes were reported in Taiwan, where the bacterial spot xanthomonads from tomato (*n* = 292) and pepper (*n* = 198) were examined over a period of 27 years (1989 to 2016) (Burlakoti et al., 2018). From 1989 to 1999, all the pepper strains (*n* = 147) and 95% of the tomato strains (*n* = 198) were identified as *X*. *euvesicatoria* pv. *euvesicatoria*. There were then transition years, from 2000 to 2009, during which 22% of tomato strains (*n* = 36) were identified as *X*. *euvesicatoria* pv. *perforans* and the remaining 78% were *X*. *euvesicatoria* pv. *euvesicatoria*. Finally, from 2010 to 2016, 92% of the pepper strains (*n* = 50) were *X*. *euvesicatoria* pv. *euvesicatoria* and 8% were *X*. *euvesicatoria* pv. *perforans*, while on tomato 99% (*n* = 58) of the strains were *X*. *euvesicatoria* pv. *perforans* (Burlakoti et al., 2018). *X. euvesicatoria* pv. *euvesicatoria* and *X*. *euvesicatoria* pv. *perforans* show evidence of extensive genome‐wide homologous recombination, and both pathovars exhibit dynamic open pan‐genomes (Jibrin et al., 2018; Newberry et al., 2019; Timilsina, Pereira‐Martin, et al., 2019). *X*. *euvesicatoria* pv. *perforans* populations in the southern USA have acquired genes from *X*. *euvesicatoria* pv. *euvesicatoria* as well as other unidentified pathovars of *X*. *euvesicatoria*, and this genomic recombination has contributed to the emergence of multiple distinct lineages of *X*. *euvesicatoria* pv. *perforans* (Newberry et al., 2019; Timilsina, Pereira‐Martin, et al., 2019). In Florida, these lineages differ in streptomycin resistance, bacteriocin production, and effector content (Klein‐Gordon et al., 2021). A unique group of *X*. *euvesicatoria* pv. *euvesicatoria* strains was isolated from tomato in Nigeria that were identical to *X*. *euvesicatoria* pv. *perforans* based on pathogenic reactions on tomato and pepper and the *hrpB2* gene sequence, but were more closely related to *X*. *euvesicatoria* pv. *euvesicatoria* based on MLSA using six housekeeping genes (Jibrin et al., 2015). Strains from Nigeria that formed a second group were phenotypically similar to *X*. *euvesicatoria* pv. *perforans*, but were more closely related to *X*. *alfalfae* pv. *allii* in the core genome (Jibrin et al., 2018). *X. euvesicatoria* pv. *perforans* strains isolated from tomato in Iran were distinct from the worldwide population of the pathogen based on the MLSA of five housekeeping genes making a novel phylogroup within the pathovar (Osdaghi, Taghavi, et al., 2018). Less information is available on the genetic diversity of *X*. *hortorum* pv. *gardneri*. Recently, MLSA showed the global distribution of a single multilocus haplotype of *X*. *hortorum* pv. *gardneri* where no genetic variation was found among strains isolated in Canada, the USA, Costa Rica, Brazil, Ethiopia, and Reunion indicating recent global spread of the bacterium (Hamza et al., 2012; Timilsina et al., 2015). Interestingly, no sequence variation was observed between the type strain of *X*. *hortorum* pv. *gardneri* isolated in 1953 in former Yugoslavia and those strains collected during the 2010s. Until recently, genetic diversity and phylogeny of *X*. *vesicatoria* have rarely been investigated. MLSA/MLST showed that a multilocus haplotype that included the type strain of *X*. *vesicatoria* was found in the strains from New Zealand and Ethiopia (Timilsina et al., 2015).

## 7 GEOGRAPHIC DISTRIBUTION

**FIGURE 3:**
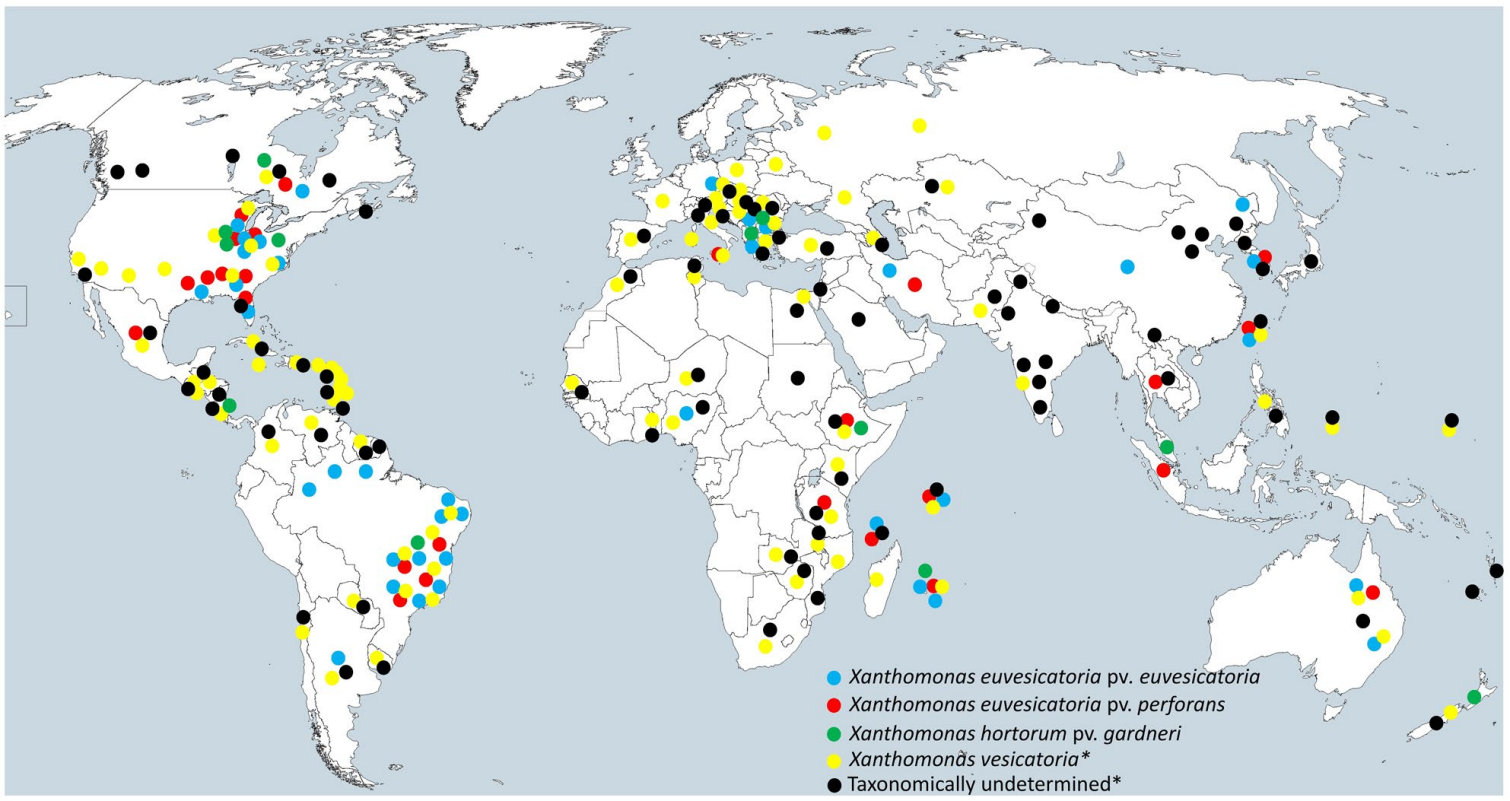
Geographic distribution of different lineages of bacterial spot xanthomonads obtained from EPPO and CABI databases up to May 2021. Due to the taxonomic complexities within tomato‐ and pepper‐pathogenic xanthomonads, the information provided in the literature before the reclassification of the pathogens in 2004 might have referred to bacterial spot as a whole (referred to as taxonomically undetermined in this map) instead of determining the species/pathovar status of the pathogens. *: According to the EPPO database (https://gd.eppo.int/taxon/XANTAV/distribution and https://gd.eppo.int/taxon/XANTVE/distribution), the pathogen may be *X*. *vesicatoria*, *X*. *euvesicatoria*, or both. The source map is from https://commons.wikimedia.org/wiki/File:A_large_blank_world_map_with_oceans_marked_in_blue.PNG

Due to the taxonomic complexities within tomato‐ and pepper‐pathogenic xanthomonads, analysis of the historic distribution of the four taxa is challenging and the current population is dynamic and changing rapidly (EPPO, 2013). The information provided in the literature before the reclassification of the pathogens in 2004 (Jones et al., 2004) might have referred to bacterial spot as a complex disease instead of determining the species/pathovar status of the pathogens. Besides the south‐eastern USA, where *X*. *euvesicatoria* pv. *perforans* prevails (Abrahamian, Klein, et al., 2019), widespread distribution of the pathogen is documented in Australia (Roach et al., 2018), Brazil (Araújo et al., 2017), Iran (Osdaghi et al., 2017), Korea (Myung et al., 2009), and Tanzania (Mbega et al., 2012). Furthermore, occurrence of *X*. *euvesicatoria* pv. *euvesicatoria* in Australia (Roach et al., 2018), Brazil (Areas et al., 2015), Bulgaria and Macedonia (Vancheva et al., 2014), Germany (Nechwatal & Theil, 2020), Iran (Osdaghi et al., 2016), Korea (Myung et al., 2015), and Tanzania (Mbega et al., 2012) is based on molecular phylogenetic evidence. As for *X*. *vesicatoria*, the pathogen prevails in Australia (Roach et al., 2018), Bulgaria (Vancheva et al., 2018), the Czech Republic (Beran et al., 2015), Egypt (Abd‐Alla & Bashandy, 2008), Ethiopia (Kebede et al., 2014), Nepal (Lamichhane et al., 2010), Tanzania (Mbega et al., 2012), and Uruguay (Cecilia et al., 2014). Until the late 20th century, *X*. *hortorum* pv. *gardneri* was rarely reported and the type strain of the pathogen was the only known strain (Bouzar et al., 1994). Since the beginning of the current century, *X*. *hortorum* pv. *gardneri* strains have increasingly been isolated in Canada, the USA, and South America and in regions bordering the Indian Ocean (Bouzar et al., 1999; Hamza et al., 2010). The global distribution of *X*. *hortorum* pv. *gardneri* has increased dramatically over the past two decades (Timilsina et al., 2015). Recent outbreaks of the bacterial spot disease in Brazil and Canada were attributed to *X*. *hortorum* pv. *gardneri* (Cândido et al., 2008). Currently, the geographic distribution of *X*. *hortorum* pv. *gardneri* has expanded from Canada to Brazil, Costa Rica (Bouzar, et al., 1999), Macedonia and Bulgaria (Kizheva et al., 2011), Ethiopia (Kebede et al., 2014), Malaysia (Rashid et al., 2016), Reunion, New Zealand (Timilsina et al., 2015), and the USA (Kim et al., 2010; Ma et al., 2011). Among the bacterial spot strains isolated in Indiana, 20% were identified as *X*. *hortorum* pv. *gardneri* (Egel et al., 2018). *X*. *hortorum* pv. *gardneri* has not been reported in the EU territories (EFSA, 2014). Figure 3 shows the global distribution of each of the bacterial spot pathogens.

## 8 BIOLOGY AND EPIDEMIOLOGY OF THE PATHOGEN

**FIGURE 4:**
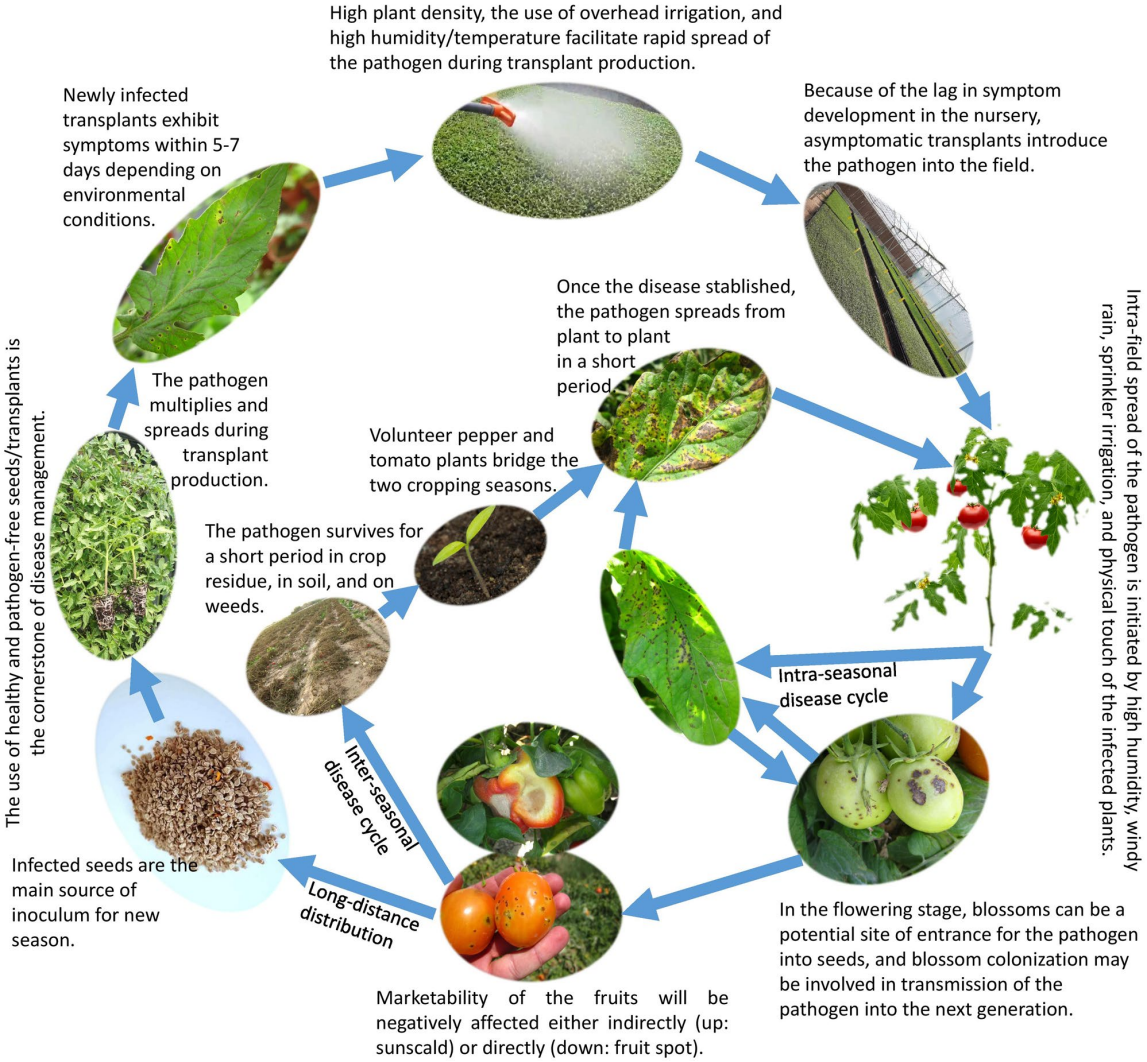
Disease cycle of bacterial spot of tomato and pepper caused by different xanthomonad lineages

Bacterial spot xanthomonads are seedborne pathogens that are primarily spread through the movement of contaminated seeds and transplants to production areas (Potnis et al., 2015). Spread of the pathogen during transplanting is of high importance (Simonton et al., 2020). Factors such as high plant densities, the use of overhead irrigation, and high humidity and temperatures facilitate rapid spread of bacterial spot xanthomonads during transplant production and can lead to severe outbreaks on seedlings (Abrahamian et al., 2021). Initial symptom development on newly infected transplants can vary from 5 to 7 days, depending on environmental conditions (Abrahamian et al., 2021). Thus, due to the lag in symptom development, transplants without symptoms can introduce the pathogen into the field. One study evaluated xanthomonad populations at two separate farm operations, finding that 60% to 100% of field strains of *X*. *euvesicatoria* pv. *perforans* were an extension of the transplant population of the pathogen (Abrahamian, Timilsina, et al., 2019). The dispersal velocity of the pathogen can be influenced by its genetic makeup. Spatiotemporal modelling of *X*. *euvesicatoria* pv. *perforans* dispersal in tomato fields demonstrated that the strains with functional T3E XopJ2 dispersed approximately three times faster than near‐isogenic mutants with nonfunctional XopJ2 under identical environmental conditions (Sharma et al., 2021). Such fitness differences affect pathogen distribution and over time may lead to directional evolution of pathogen populations in which the strains carrying such genes are enriched (Sharma et al., 2021). Once the disease is established on a plant in the flowering stage, blossoms can be a potential site of entrance for the pathogen into seeds, and blossom colonization may be involved in transmission of the pathogen into the next generation (Dutta et al., 2014). Figure 4 illustrates the bacterial spot disease cycle from different points of view, where environmental conditions (temperature and humidity), infested plant debris, and infected seed lots play determinative roles in the intraseasonal, interseasonal, and long‐distance distribution of the pathogen, respectively. Seed contamination levels in pepper infected with *X*. *euvesicatoria* pv. *euvesicatoria* were reported to range from 34 to 100 cfu/g (Giovanardi et al., 2018). Successful transmission of *X*. *euvesicatoria* pv. *euvesicatoria* in pepper seeds has been recorded in 16% of the seed lots while the pathogen was detected in 39% of the pepper seed lots and viable colonies were recovered from 35% of the seeds. A positive correlation was observed between the inoculum concentration of the pathogen on pepper blossoms and the percentage of infested seed lots (Dutta et al., 2014). Bacterial spot severity may be influenced by the level of macronutrient and micronutrient concentrations in the soil, affecting the expression of plant disease resistance genes in the systemic acquired resistance (SAR) pathway (Dutta et al., 2017). Plant‐pathogenic xanthomonads can survive on taxonomically diverse plant species other that their host plants in natural conditions (Zarei et al., 2018). The bacterial spot pathogen's ability to survive between crops on volunteer pepper and tomato plants, and for short periods in crop residue in soil and on weeds, is also worthy of management consideration, although their relative epidemiological contribution to seasonal outbreaks compared to the other inoculum sources remains undetermined (Jones et al., 1986; Santos et al., 2020; Stall et al., 2009).

## 9 PATHOGENICITY MECHANISMS

### 9.1 Type II secretion system

Two type II secretion systems (T2SSs), Xcs and Xps, have been identified in xanthomonads. Szczcsny et al. (2010) mutated *xcsD* and *xpsD* (both encode predicted outer membrane secretins) in Xcs and Xps, respectively, and determined that Xps contributes towards pathogenesis or overall pathogen fitness, whereas Xcs does not affect virulence. They also demonstrated that the Xps system contributes to protease and xylanase activity. However, they determined that Xcs is involved in the interplay between these secretion systems and the type III secretion system (T3SS).

### 9.2 Type III secretion system and effectors

With the advances in the area of molecular biology over the last few decades, our understanding of molecular mechanisms in plant‐pathogenic bacteria has rapidly evolved. Much of our understanding of the T3SS and its regulation in xanthomonads has resulted from studies involving *X*. *euvesicatoria* by Ulla Bonas and her colleagues in Germany. The first *hrp* gene cluster in a xanthomonad was identified in *X*. *campestris* pv. *vesicatoria*. It contains six transcription units, designated *hrpA* to *hrpF* (Bonas et al., 1991). Two regulators of the T3SS, HrpG and HrpX, in *X*. *euvesicatoria* regulate Hrp expression in plant tissue (Wengelnik & Bonas, 1996; Wengelnik et al., 1996).

Several of the earliest described T3Es in xanthomonads were identified in *X*. *euvesicatoria*. Following the creation of near‐isogenic lines of Early Calwonder (ECW) carrying resistance genes *Bs1*, *Bs2*, or *Bs3*, at the University of Florida by Robert Stall, these lines were used to identify clones carrying *avrBs1*, *avrBs2*, or *avrBs3*, respectively, and also importantly to demonstrate the gene‐for‐gene response in bacteria (Minsavage et al., 1990). Much of this work was done by Brian Staskawicz and collaborators at the University of California – Berkeley. Using Southern hybridization with an *avrBs2* probe, the gene was one of the first T3E genes to be identified in a wide range of xanthomonads (Kearney & Staskawicz, 1990). Furthermore, the gene was cloned from diverse xanthomonads and expressed in *X*. *euvesicatoria*, with many of them eliciting a hypersensitive reaction (HR) in ECW carrying *Bs2* (ECW20R). Through mutagenesis and complementation experiments, *avrBs2* was determined to contribute to fitness. The C‐terminal half of AvrBs2 had highest homology with enzymes synthesizing or hydrolysing phosphodiester linkages and in particular with agrocinopine synthase (Swords et al., 1996). Although there was speculation that incorporation of *Bs2* into pepper varieties would potentially provide durable resistance based on *avrBs2* mutants being less virulent (Kearney & Staskawicz, 1990), naturally occurring mutant *X*. *euvesicatoria* strains as well as those isolated from lesions that developed on ECW20R in controlled experiments that did not elicit an HR in ECW20R had reduced fitness, unlike the strain complemented with a wild‐type *avrBs2* (Swords et al., 1996). Sequence analysis of the *avrBs2* genes from these mutant strains that grew to moderately high populations in ECW20R revealed two types of mutations in *avrBs2*, a 5‐bp insertion or a divergent 3′ region, that reduced fitness. With the mutant strains carrying variant forms of *avrBs2* being nonfunctional in ECW20R and also having reduced virulence, *Bs2* was considered a potential source of durable resistance. However, *X*. *euvesicatoria* strains were isolated in the mid to late 1990s from lesions on *Bs2* pepper; sequence analysis of several *avrBs2* genes revealed point mutations that led to single amino acid changes (Gassmann et al., 2000). Unlike mutations identified by Swords et al. (1996), the point mutations resulting in single amino acid changes had minimal effects on fitness. The results show that *avrBs2* has evolved to avoid recognition by *Bs2* and thus maintain a higher level of virulence.

The *avrBs3* gene was originally cloned by Bonas et al. (1989) and was determined to be present on the self‐transmissible plasmid pXV11 in *X*. *euvesicatoria* strain 71‐21. This gene has been designated as the type member of TALEs (Boch & Bonas, 2010). Southern hybridization with an *avrBs3* probe revealed significant homology in other xanthomonads, including the cotton pathogen *X*. *axonopodis* pv. *malvacearum*. Sequence analysis of *avrBs3* revealed it to be unique among *avr* genes in that it contains 17.5 near‐identical repeats, each of which consists of 102 base pairs with the repeat region being present in the centre of the gene (Bonas et al., 1989). Following identification of *avrBs3*, homologs including *avrBs3*‐2, later known as *avrBs4*, which interacts with *Bs4* in tomato, and *avrHah1*, which interacts with *Bs3*, were identified (Bonas et al., 1993; Schornack et al., 2004, 2008). *AvrBs4* elicits an HR in tomato and also in *C. pubescens*, which carries *Bs4C* (Strauss et al., 2012). An *avrBs3* probe was used to identify two TALEs in the rice pathogen *Xanthomonas oryzae* (Hopkins et al., 1992). After identification of *avrBs3* it was determined that the repeat region was responsible for host specificity following deletion of individual repeats followed by inoculation of tomato and different pepper genotypes (Herbers et al., 1992). Following cloning of *Bs3* from pepper it was determined that expression of the gene was associated with the binding of AvrBs3 to a specific DNA element (UPA box) in the *Bs3* promoter (Römer et al., 2007, 2009). Identification of the UPA box and the fact that it is roughly equal to the number of repeats in *AvrBs3* were important factors in breaking of the code (Boch & Bonas, 2010; Boch et al., 2009; Moscou & Bogdanove, 2009).

The XopJ effector family was identified as a family of effectors in *X*. *euvesicatoria* pv. *euvesicatoria* and *X*. *euvesicatoria* pv. *perforans* strains. In addition to the three *avr* genes that elicit an HR in pepper differentials containing *Bs1*, *Bs2*, or *Bs3* identified in the study by Minsavage et al. (1990), *avrBsT* (*xopJ2*) was also identified to be present in tomato strains and shown to elicit an HR in pepper. Whalen et al. (1993) identified *avrRxv* (*xopJ4*) in an *X*. *euvesicatoria* pv. *euvesicatoria* strain that was previously shown to elicit an HR in the tomato genotype Hawaii 7998 (Jones & Scott, 1986). Astua‐Monge et al. (2000) identified *avrXv4* (XopJ4) in *X*. *euvesicatoria* pv. *perforans*, which when expressed in the bacterium was responsible for eliciting an HR on *Solanum pennelli* (Astua‐Monge et al., 2000).

Availability of genome sequences of multiple strains led to the identification of several putative effectors based on sequence homologies and based on the identification of conserved domains or motifs using machine learning approaches. These and others with inferred functions in plant pathogenesis have been reviewed in Potnis et al. (2015). Here we discuss advances in effector biology in the recent six years. Teper et al. (2018) used a machine learning approach to identify seven novel effectors, XopAU, XopAV, XopAW, XopAP, XopAX, XopAK, and XopAD, in *X*. *euvesicatoria* pv. *euvesicatoria* strain 85‐10 and subsequently confirmed their translocation into the plant cell. A novel enzymatic activity was documented for one of these effectors, XopAU, which is a catalytically active serine/threonine protein kinase that manipulates host immune signalling through the direct phosphorylation of the MAPK receptor MKK2 (Teper et al., 2016). It remains undetermined how the phosphorylation of MKK2 facilitates the infection process. The expression of XopAU contributes to symptom development in pepper leaves but not bacterial growth, which is a common feature of many T3Es used by *X*. *euvesicatoria* pv. *euvesicatoria* and is probably the result of their functional redundancy. For example, approximately half of the effector proteins (17 out of 33) secreted by *X*. *euvesicatoria* pv. *euvesicatoria* strain 85‐10 serve to suppress pattern‐triggered immunity through various mechanisms, suggesting a primary role for this class of virulence factor (Popov et al., 2016). An emerging target of some effector proteins includes the plant microtubule network. The effector XopL, which was initially characterized for its role as an E3 ubiquitin ligase that interacts with the plant ubiquitination system (Singer et al. 2013), also colocalizes with microtubules independent of its enzymatic activity (Erickson et al., 2018). This interaction leads to the suppression of stromule formation as well as the transfer of plastids to the nucleus. Likewise, AvrBsT acetylates a microtubule‐associated protein (ACIP1) in *Arabidopsis* that is essential for the plant immune response. This causes ACIP1 to form aggregates throughout the plant cell and alter its interaction with the microtubule network in a fashion that remains to be elucidated. Moreover, AvrBsT recognition also leads to the accumulation of phosphatidic acid, a lipid signal that coincides with effector‐triggered immunity and alters cytoskeletal components through direct binding of tubulin and the microtubule‐bundling protein MAP65‐1 (Zhang et al., 2012).

TALEs also contribute significantly to the fitness of bacterial spot xanthomonads but are not considered major virulence determinants as they are in other *Xanthomonas* spp. (reviewed by Boch & Bonas, 2010; Khojasteh et al., 2020). Three TALEs, AvrBs3, AvrBs4, and AvrHah1, are commonly found in *X*. *vesicatoria*, *X*. *hortorum* pv. *gardneri*, and *X*. *euvesicatoria* pv. *euvesicatoria*. However, TALEs were only recently documented in emerging lineages of *X*. *euvesicatoria* pv. *perforans* (Jibrin et al., 2018; Newberry et al., 2019). One of these is AvrHah1, which was originally described in *X*. *hortorum* pv. *gardneri* (Schornack et al., 2008) and serves to up‐regulate two basic helix–loop–helix (bHLH) transcription factors in tomato and pepper, which in turn promote the expression of plant pectate lyase genes (Schwartz et al., 2015). This leads to profuse water‐soaking of the leaf tissue and probably facilitates pathogen ingress and/or dispersal under field conditions. Likewise, PthXp1 is a new class of TALEs identified in *X*. *euvesicatoria* pv. *perforans* (Newberry et al., 2019). This TALE serves as a virulence factor through promoting chlorosis and symptom development in tomato leaves, while escaping recognition of the *Bs3* and *Bs4* genes, which together confer resistance (Minsavage et al., 1990) to the suite of TALEs used by the *Xanthomonas* spp. associated with tomato and pepper.

### 9.3 Other virulence strategies

While *Xanthomonas* spp. rely on the T2SS and the T3SS to inject virulence factors into the extracellular milieu and the plasma membrane, they may also use alternative transport routes. Small RNAs were identified in *X*. *euvesicatoria* and one, sX12, when mutated in the bacterium resulted in the bacterium having reduced virulence and delay in HR (Schmidtke et al., 2012). Translocation of the TALE AvrBs3 by *X*. *euvesicatoria* pv. *euvesicatoria* occurs in vivo during the early stages of the infection process, before the activation of HpaB, which serves as the translocon for effector proteins through the T3SS apparatus (Scheibner, Hartmann, et al., 2017). Similarly, many extracellular degrading enzymes that promote the growth and virulence of *X*. *euvesicatoria* pv. *euvesicatoria* are delivered through outer membrane vesicles in addition to the T2SS (Solé et al., 2015). Various virulence factors other than T3Es, including adhesins, lipopolysaccharides, cell wall‐degrading enzymes, and the regulatory network involved in coordination of virulence factors during pathogenesis, were extensively reviewed by Buttner and Bonas (2010). Their review demonstrated the intricate network involving two‐component systems and transcriptional regulators, Clp, Zur, HpaR, and HrpX, as well as posttranscriptional regulators such as RsmA. Recent studies by Buttner's group of the regulation of T3Es have revealed that there is hierarchical secretion and translocation of T3Es into plant cells during the infection process. The T3S chaperone HpaB recognizes the translocation signal and acts as an escort protein (Scheibner, Marillonnet, et al., 2017). Buttner's group has further dissected interactions of various components of T3SS proteins and found that HrcVc interacts with the early substrate HrpB2, the pilus protein HrpE, and other T3SS components and is important in substrate docking (Hartmann & Buttner, 2013). Interestingly, interactions of HrcU helped resolve an important question about the control of translocation of different substrates, such as early or late effectors. The switch in substrate specificity involves cleavage and release of HrcU bound to the early substrates HrpB2 and HpaC (Hausner & Buttner, 2014).

## 10 OTHER MECHANISMS ASSOCIATED WITH PLANT–MICROBE INTERACTIONS

In the bacterial spot pathogens, different genes are activated in response to the changing environment to enable them to survive, adapt, evade host defences, propagate, and damage the host (Tamir‐Ariel et al., 2007). Felipe et al. (2018) evaluated several characteristics involved in virulence and strain aggressiveness: motility, biofilm formation, adhesion, and production of xanthan. They noted that the most aggressive strains exhibited the greatest swarming and twitching motilities and developed a mature biofilm with a homogeneous and compact structure and higher biomass and substratum coverage than the other strains. Recombinase‐based in vivo expression technology was implemented to study the genes activated in the bacterial spot pathogen during its interaction with tomato. The technique revealed 61 unique *X*. *campestris* pv. *vesicatoria* genes or operons that delineate a picture of the different processes involved in pathogen–host interactions (Tamir‐Ariel et al., 2007). It has been shown that the aggressiveness of *X*. *vesicatoria* is related to its ability to move by flagella or type IV pili, adhere to leaves, and form well‐developed biofilms, factors that improve phyllosphere colonization (Felipe et al., 2018). The roles that type IV pili play in *Xanthomonas* pathogenesis vary from species to species (Dunger et al., 2016; Shah et al., 2021). In *X*. *campestris* pv. *vesicatoria*, the *fimA* mutant exhibited dramatically reduced cell aggregation in laboratory cultures and on infected tomato leaves. The *fimA* mutant strain also exhibited decreased tolerance to ultraviolet light (Ojanen‐Reuhs et al., 1997). Pilus extension and retraction is regulated by cyclic‐di‐GMP‐binding regulatory protein complexes (Dunger et al., 2016).

## 11 DETECTION, ISOLATION, AND IDENTIFICATION OF THE PATHOGEN

Recently, Miller et al. (2017) provided a detail‐oriented detection protocol for *Xanthomonas* spp. in tomato and pepper seeds. Beside the conventional specific/semispecific culture media, serological techniques, DNA fingerprinting methods, and highly specific and sensitive PCR‐based techniques are available for the detection of bacterial spot pathogens (Leite et al., 1995; Pečenka et al., 2020). Simultaneous occurrence of bacterial spot and bacterial canker (caused by *Clavibacter michiganensis*) pathogens in the same tomato field has been recorded (Ansari et al., 2019; Osdaghi, Ansari, et al., 2018). Both of these pathogens as well as a number of tomato‐associated nonpathogenic bacteria, for example, epiphytic *Curtobacterium flaccumfaciens* strains (Osdaghi, Taghavi, Hamzehzarghani, et al., 2018), produce yellow‐pigmented, domed circular mucoid colonies on culture media, making the initial identification of the causal agent challenging. The primer pairs Bs‐XeF/Bs‐XeR, Bs‐XvF/Bs‐XvR, Bs‐XgF/Bs‐XgR, and Bs‐XpF/Bs‐XpR were designed for specific detection and discrimination of the four bacterial spot xanthomonads *X*. *euvesicatoria* pv. *euvesicatoria*, *X*. *vesicatoria*, *X*. *hortorum* pv. *gardneri*, and *X*. *euvesicatoria* pv. *perforans*, respectively (Koenraadt et al., 2009), although the expected 197‐bp DNA fragment has not been amplified using the primer pair Bs‐XpF/Bs‐XpR in the *X*. *euvesicatoria* pv. *perforans* strains isolated in Iran, questioning the usability of these primers in all geographic areas (Osdaghi et al., 2017). Sensitivity of the primers in conventional PCR is 50 pg/µl for purified DNA and ranges from 5 × 10^2^ to 5 × 10^4^ cfu/ml in bacterial suspensions (Araújo, Costa, et al., 2012). A multiplex real‐time TaqMan PCR assay based on a 420‐bp fragment of the *hrpB7* gene is available for simultaneous detection and discrimination of the four bacterial spot pathogens using a specific probe for each lineage (Strayer, Jeyaprakash, et al., 2016). A highly specific recombinase polymerase amplification method has also been developed for in‐field detection of bacterial spot pathogens. The technique is isothermal, rapid, and more tolerant against inhibitors compared to PCR (Strayer‐Scherer et al., 2019). Furthermore, a combination of three restriction endonucleases (*Alu*I, *Mbo*I, and *Hpa*II) via 16S‐23S internal transcribed spacer ribosomal DNA PCR–restriction fragment length polymorphism analysis successfully differentiated the four lineages of bacterial spot pathogen (Kizheva et al., 2016). New technologies including ultraviolet, visible, and near‐infrared reflectance spectroscopy were used to diagnose the bacterial spot and determine disease severity on tomato (Borgeset al., 2016; Jones et al., 2010).

The primer pairs ZnDoF/ZnDoR and Xeu2.4/Xeu2.5 amplify 100‐ and 208‐bp DNA fragments, respectively, in *X*. *euvesicatoria* pv. *euvesicatoria* strains but not in the other three bacterial spot pathogens (Moretti et al., 2009; Pečenka et al., 2020). Furthermore, the unique gene *recG* has been used to design primers for a loop‐mediated isothermal amplification assay to rapidly and accurately identify and differentiate *X*. *euvesicatoria* pv. *euvesicatoria* from other bacterial spot‐causing *Xanthomonas* spp. using a field‐deployable portable BioRanger instrument (Larrea‐Sarmiento et al., 2018). The primer pairs XV1F/XV1R and Xv‐gyrB‐F/Xv‐gyrB‐R direct the amplification of 365‐ and 104‐bp DNA fragments, respectively, in *X*. *vesicatoria* strains capable of differentiating this species from the other three pathogens (Araújo et al., 2013; Beran & Mráz, 2013). *X. hortorum* pv. *gardneri* is distinguishable from the other three lineages using the GENIII 96 microplate (Biolog), which provides reliable, accurate identification of the suspect strains based on 94 phenotypic tests (Stoyanova et al., 2014). Furthermore, MLSA using the sequences of six housekeeping genes (*fusA*, *gapA*, *gltA*, *gyrB*, *lacF*, and *lepA*) and the phylogeny of *hrpB2* is a reliable method for identification and discrimination of the bacterial spot xanthomonads from closely related pathogens (Hamza et al., 2012; Obradovic et al., 2004; Osdaghi, Taghavi, et al., 2018; Timilsina et al., 2015).

## 12 MANAGEMENT

Bacterial spot is best managed early during the production cycle, beginning with the use of healthy and pathogen‐free seeds and transplants to exclude the pathogen, avoiding the handling of wet plant material and free moisture on foliage to prevent disease development and spread, as well as the application of protective chemicals or biological treatments to reduce the severity and spread during transplant production (Abrahamian, Jones, et al., 2019; Potnis et al., 2015). It is recommended to rogue transplants with symptoms to eliminate primary inoculum sources while rogueing the transplants without symptoms as far as 1–3 m away from diseased plants, depending on environmental conditions, to reduce the introduction of infected transplants into the field (Abrahamian et al., 2021). Approved protective options to limit subsequent spread and disease development during open‐field production are limited, especially when environmental conditions favour rapid disease development. The effectiveness of many protective chemicals and biological treatments can vary greatly due to many factors, such as rain fastness, application timing, bactericide tolerance, and the rate of disease development. Even under ideal conditions, effective applications can have relatively little benefit to yield, further questioning the role of defoliation alone in yield reductions.

### 12.1 Biological control

The window of infection for bacterial spot is quite long (from the seed stage to fruit harvesting), making biological control of the disease challenging as either antagonists or their products need to be sustained throughout the infection period. Several biological control approaches are available for the management of bacterial spot, while only few are tested for their applicability in field conditions (Gasic et al., 2018). For instance, foliar application of tailocins (phage‐tail‐like bacteriocins produced by *Pseudomonas fluorescens* SF4c) against *X*. *vesicatoria* and *Bacillus velezensis* GF267 against *X*. *euvesicatoria* pv. *perforans* reduces the disease severity and incidence in the greenhouse (de Paula Kuyat Mates et al., 2019; Príncipe et al., 2018). The plant growth‐promoting rhizobacterium (PGPR) *Bacillus pumilus* S2‐3‐2 and a mixture of *Bacillus* spp. reduce bacterial leaf spot severity by eliciting SAR (Liu et al., 2018). The PGPR *Bacillus* sp. DFs1420 reduced *X*. *hortorum* pv. *gardneri* disease severity by 48% in tomato (Naue et al., 2014). *Rahnella aquatilis* application on seed, soil, root, or leaves reduced *X*. *euvesicatoria* pv. *euvesicatoria* incidence in tomato (Al‐Dahmani et al., 2003). Foliar application of a combination of the antagonist *Pseudomonas* *syringae* strain Cit7 and the PGPR *P*. *fluorescens* 89B‐61 significantly reduced bacterial leaf spot on tomato in the field (Ji et al., 2006). In addition to bacterial biocontrol agents, various fungi also showed biocontrol potential against *X*. *euvesicatoria* pv. *euvesicatoria*, mostly by producing nonvolatile metabolites (Casaroto et al., 2017).

Integration of biological control agents and SAR inducers (harpin and acibenzolar‐*S*‐methyl [ASM]) increases the efficiency of bacterial spot management (Abo‐Elyousr & El‐Hendawy, 2008; Obradovic et al., 2005). Wettable powder of *Bacillus subtilis* QST 713 tank mixtures with copper hydroxide reduce bacterial leaf spot severity in tomato (Abbasi & Weselowski, 2015). Application of specific bacteriophages alone or in combination with biocontrol agents and/or copper hydroxide significantly reduces disease incidence (Balogh et al., 2018; Gasic et al., 2018; Jones et al., 2012; Ríos‐Sandoval et al., 2020). However, bacteriophages often degrade when exposed to ultraviolet light, leading to a major challenge under field conditions (Iriarte et al., 2007). Furthermore, there is no correlation between disease control efficacy and in vitro phage multiplication, in vitro bacterial suppression, or in vivo phage multiplication in the presence of the host (Balogh et al., 2018). In most field trials a single biological agent is not effective but has synergistic effects with chemical control measures. Šević et al. (2019) designed an efficient integrated disease management programme where integration of copper hydroxide, the SAR inducer ASM, and bacteriophage strain KФ1 was capable of reducing the disease severity by 96%–98%. *X. euvesicatoria* pv. *perforans* 91‐118 produces at least three different bacteriocin‐like compounds (BCN‐A, BCN‐B, and BCN C) and has antagonistic activity against *X*. *euvesicatoria* pv. *euvesicatoria* strains (Hert et al., 2005; Tudor‐Nelson et al., 2003). A bacteriocin‐producing strain of *X*. *euvesicatoria* pv. *perforans* with attenuated pathogenicity was successfully applied for biocontrol of a bacteriocin‐sensitive strain of *X*. *euvesicatoria* pv. *euvesicatoria* (Hert et al., 2009). Compost extract reduced bacterial leaf spot severity in transplants, but in field conditions neither foliar spray nor combination with soil amendment was able to suppress the disease (Al‐Dahmani et al., 2003).

### 12.2 New horizons in chemical control of the bacterial spot

The most common approach for management of bacterial spot pathogens is the preventive application of copper‐based bactericides, either alone or in combination with ethylene‐*bis*‐dithiocarbamate fungicides and antibiotics. However, consistent control of the disease is challenging when optimal environmental conditions for development of bacterial spot is present (Vallad et al., 2010) while occurrence of copper tolerance/resistance in populations of bacterial spot pathogens often contributes to poor field control of the disease (Abbasi et al., 2015; Araújo, Pereira, et al., 2012; Khanal et al., 2020; Martin et al., 2004). Interestingly, copper tolerance in bacterial plant pathogens was first described in *X*. *euvesicatoria* pv. *euvesicatoria* (Marco & Stall, 1983). The resistance was associated with a self‐transmissible plasmid that carried the copper resistance genes (Stall et al., 1986). Overreliance on copper‐based chemicals in agriculture has resulted in environmental and ground water pollution (Lamichhane et al., 2018). Recently, Griffin et al. (2017) provided a comprehensive review on copper resistance in bacterial spot pathogens of tomato and pepper. During the past decade, there has been an increasing trend in the number of new biological or chemical products to substitute copper‐based compounds in bacterial spot management. The new copper composites coreshell copper (CS‐Cu), multivalent copper (MV‐Cu), and fixed quaternary ammonium copper (FQ‐Cu) have shown promising results as potential alternatives to commercially available micron‐sized copper bactericides (Strayer‐Scherer et al., 2018). Greenhouse assays using three copper‐based nanocomposites gave promising results, while MV‐Cu is the only copper composite with no phytotoxicity on plants under controlled conditions (Fan et al., 2021).

Nanoparticles of magnesium oxide (Nano‐MgO) gave promising results in field experiments (Liao et al., 2019a). Also, a hybrid nanoparticle of copper–zinc (Cu/Zn) showed promising results in controlling bacterial spot in greenhouse conditions (Carvalho et al., 2019). Doped zinc‐oxide nanocrystals also showed promising results in control of the disease (Fraga et al., 2021). Application of photocatalytic nanoscale titanium dioxide (TiO_2_), nanoscale TiO_2_ doped with silver (TiO_2_/Ag), and nanoscale TiO_2_ doped with zinc (TiO_2_/Zn; AgriTitan) has also provided promising results (Paret et al., 2013). In greenhouse studies, tomato plants treated with silver‐based nanocomposite Ag‐dsDNA‐GO showed significantly lower disease severity when compared to copper–mancozeb (Strayer, Ocsoy, et al., 2016, 2016). Furthermore, application of ASM, copper octanoate, quinoxyfen, oxysilver nitrate, and pentasilver hexaoxoiodate significantly reduced disease severity on tomato transplants and increased field production (Abrahamian, Jones, et al., 2019). A synergistic interaction between copper hydroxide, cymoxanil, and famoxadone (components of Tanos 50DF) was observed in reducing the growth of a copper‐resistant strain of *X*. *euvesicatoria* pv. *perforans* (Fayette et al., 2012; Roberts et al., 2008).

Foliar spray applications of a commercial chitosan extract (Armour‐Zen) as well as *N*‐acetylcysteine amended with copper significantly reduced the incidence of bacterial spot in tomato (Coqueiro & Di Piero, 2011; Qiao et al., 2021; Ramkissoon et al., 2016). Carvacrol (a plant‐derived small molecule) increased the sensitivity of a copper‐resistant *X*. *euvesicatoria* pv. *perforans* strain to copper (Qiao et al., 2020). It has been shown that 2,6‐dichloroisonicotinic acid enhances the expression of defence genes in tomato seedlings against *X*. *euvesicatoria* pv. *perforans* (Chandrashekar & Umesha, 2014). The new compound 3‐indolylacetonitrile significantly reduces bacterial spot on tomato and enhances the efficacy of copper‐based chemicals, for example, Kocide 3000, against the pathogen (Liu et al., 2019). Two random peptide mixture compounds (random combination of l‐phenylalanine and l‐ or d‐lysine along the 20‐mer chain length of the peptides) as well as plant activators, for example, ASM, that trigger SAR against the bacterial spot pathogen gave promising results in field conditions (Pontes et al., 2016; Topman et al., 2018).

### 12.3 Novel achievements in host resistance development

Several attempts have been made to develop tomato and pepper lines possessing resistance to bacterial spot xanthomonads (Zhao et al., 2015; Bernal et al., 2020; Bhattarai et al., 2017; Kunwar et al., 2018; Li et al., 2019; Liabeuf, 2016; Liabeuf et al., 2015, 2018; Potnis et al., 2019; Scott et al., 2015; Sim et al., 2015; Timilsina et al., 2016; Wang et al., 2017). Stall et al. (2009) reviewed the availability of resistant cultivars and the durability of resistance in tomato and pepper against xanthomonads causing bacterial spot. Determination of virulence properties and race differentiation of the pathogen in a given geographic area is a prerequisite for development of resistant lines (Damicone et al., 2020; Jibrin et al., 2018; Klein‐Gordon et al., 2021; Timilsina et al., 2016; Wang et al., 2018). A number of quantitative trait loci (QTLs) for resistance against bacterial spot pathogens have been described in the literature (Liabeuf et al., 2018). Using QTL mapping, three independent sources of resistance to bacterial spot pathogens in the centromeric region on chromosome 11 derived from tomato line Hawaii 7998 (QTL‐11A), PI 114490 (QTL‐11B), and LA2533 (QTL‐11C) have been identified (Bernal et al., 2020). Whole genome sequence‐based investigations of effector profiles in *X*. *euvesicatoria* pv. *perforans* populations collected between 1991 and 2012 showed that XopJ4 and AvrBsT are the best targets for resistance breeding against bacterial spot in tomato (Timilsina et al., 2016). Furthermore, genome‐wide association studies provide alleles that could be used for resistance gene pyramiding against the pathogens (Potnis et al., 2019). Resistance to *X*. *euvesicatoria* pv. *perforans* race T4 in tomato breeding lines was reported by Bhattarai et al. (2017). Stall et al. (2009) described the progress in the cloning of avirulence genes and identification of resistance‐related genes in tomato and pepper over the past several decades.

## 13 CONCLUSIONS AND FUTURE AVENUES FOR RESEARCH

Since the first description of bacterial spot in 1920, different aspects of the pathogens, that is, biology, epidemiology, and plant–microbe interactions, have been extensively studied, providing the fundamentals of knowledge required for effective disease management. More specifically, we have a profound understanding of the factors influencing pathogen dispersal, transmission, and disease outbreaks. Furthermore, the role of quarantine inspections, crop sanitary control, and resistant cultivars in the management of bacterial spot disease is highlighted in the literature, while the use of pathogen‐free high‐quality seed lots is the cornerstone of disease management. Recent technological advancements in high‐throughput DNA sequencing have provided a giant step forward in our understanding of virulence properties and pathogenicity factors of the bacterial spot xanthomonads. Advanced technologies allow us to predict and test which genes are involved in the pathogenicity of the pathogens, providing opportunities to develop new resistant tomato and pepper lines against the pathogens, and at the same time enabling us to develop state‐of‐the‐art genome‐informed detection methods to trace seed infections with lower efforts and cost. Finally, recent achievements in the study of host–pathogen interactions ensure that in the coming years we will integrate all discoveries into a comprehensive understanding of the biology, genomics, and virulence properties of bacterial spot pathogens to pave the way for research on the sustainable management of the disease in the 21st century.

## CONFLICT OF INTEREST

The authors declare that the research was conducted in the absence of any commercial or financial relationships that could be construed as a potential conflict of interest.

## AUTHOR CONTRIBUTION

E.O. conceived and designed the work with assistance from J.B.J. A.S. and E.O. designed and illustrated the graphics and figures. All coauthors contributed to writing different sections of the manuscript under the supervision of J.B.J.

## Data Availability

Data sharing is not applicable to this article as no new data were created or analysed.
